# Changes in the transcriptome, ploidy, and optimal light intensity of a cryptomonad upon integration into a kleptoplastic dinoflagellate

**DOI:** 10.1038/s41396-020-0693-4

**Published:** 2020-06-08

**Authors:** Ryo Onuma, Shunsuke Hirooka, Yu Kanesaki, Takayuki Fujiwara, Hirofumi Yoshikawa, Shin-ya Miyagishima

**Affiliations:** 1 Department of Gene Function and Phenomics, National Institute of Genetics, Yata 1111, Mishima, Shizuoka 411-8540 Japan; 2 Research Institute of Green Science and Technology, Shizuoka University, 836 Ohya, Suruga, Shizuoka 422-8529 Japan; 3 Department of Genetics, Graduate University for Advanced Studies (SOKENDAI), Yata 1111, Mishima, Shizuoka 411-8540 Japan; 4 Department of Bioscience, Tokyo University of Agriculture, 1-1-1 Sakuragaoka, Setagaya, Tokyo, 156-8502 Japan

**Keywords:** Cellular microbiology, Transcriptomics

## Abstract

Endosymbiosis of unicellular eukaryotic algae into previously nonphotosynthetic eukaryotes has established chloroplasts in several eukaryotic lineages. In addition, certain unicellular organisms in several different lineages ingest algae and utilize them as temporal chloroplasts (kleptoplasts) for weeks to months before digesting them. Among these organisms, the dinoflagellate *Nusuttodinium aeruginosum* ingests the cryptomonad *Chroomonas* sp. and enlarges the kleptoplast with the aid of the cryptomonad nucleus. To understand how the cryptomonad nucleus is remodeled in the dinoflagellate, here we examined changes in the transcriptome and ploidy of the ingested nucleus. We show that, after ingestion, genes involved in metabolism, translation, and DNA replication are upregulated while those involved in sensory systems and cell motility are downregulated. In the dinoflagellate cell, the cryptomonad nucleus undergoes polyploidization that correlates with an increase in the mRNA levels of upregulated genes. In addition, the ingested nucleus almost loses transcriptional responses to light. Because polyploidization and loss of transcriptional regulation are also known to have occurred during the establishment of endosymbiotic organelles, these changes are probably a common trend in endosymbiotic evolution. Furthermore, we show that the kleptoplast and dinoflagellate are more susceptible to high light than the free-living cryptomonad but that the ingested nucleus reduces this damage.

## Introduction

Several nonphotosynthetic unicellular eukaryotic lineages are capable of accommodating various lineages of algal endosymbionts or chloroplasts sequestrated from algae (known as kleptoplasts) that are ingested by phagocytosis [1]. Such acquired phototrophy is widespread in aquatic ecosystems although it is generally more frequent in oligotrophic environments [2]. In certain cases, organisms that undergo this process become major primary producers; these include red tide ciliates, which obtain kleptoplasts derived from a cryptophyte prey, and some benthic foraminifera, which host several lineages of algae [1].

The process of acquired phototrophy is also believed to have contributed to multiple independent acquisitions of chloroplasts (and nonphotosynthetic plastids) by previously nonphotosynthetic eukaryotic lineages [3, 4]. More than a billion years ago, the nonphotosynthetic eukaryotic ancestor of Archaeplastida acquired a chloroplast through primary endosymbiosis with a cyanobacterium giving rise to glaucophytes, red algae, and Viridiplantae (green algae and plants). Chloroplasts were then further spread into other eukaryotic lineages through secondary and tertiary endosymbiotic events in which a eukaryotic algae was integrated into a heterotrophic eukaryote. The chloroplasts of red algae were distributed to stramenopiles, dinoflagellates, apicomplexans, chromerids, haptophytes, and cryptophytes, whereas those of green algae were obtained by euglenophytes and chlorarachniophytes. In addition, some species of dinoflagellate further replaced their original chloroplast of red algal origin with those of green algae, cryptophytes, haptophytes, or diatoms [3].

Among the acquired phototrophic organisms, those that are kleptoplastic exhibit different degrees of similarity with those possessing chloroplasts of secondary or tertiary endosymbiotic origin depending on the species as follows [1]. Kleptoplastic organisms have been reported in a wide range of eukaryotic lineages such as ciliates [5], dinoflagellates [6–9], a katablepharid [10], foraminiferans [11], and metazoans [12, 13]. Some species temporally retain kleptoplasts but immediately digest the nucleus of algal prey after ingestion. In contrast, the nucleus of algal prey is temporally retained with the kleptoplast in other species, including the ciliate *Mesodinium rubrum*, some dinoflagellates, the katablepharid *Hatena arenicola*. In addition, among the dinoflagellates *Durinskia* spp., *Durinskia capensis* temporally retains the kleptoplast and nucleus of a diatom prey, whereas two other species permanently retain diatom cells in which the diatom nucleus and chloroplasts divide in accordance with the cell division of the dinoflagellate host [14, 15]. The retention of the endosymbiont nucleus and the chloroplast division in accord with the host cell cycle are also observed in cryptophytes and chlorarachniophytes, which possess bona fide chloroplasts with a vestigial nucleus of red and green algal origin, respectively, but with a greatly reduced genome size [16, 17].

Generally, kleptoplastic organisms that do not retain their prey’s nucleus are mixotrophic, whereas those that retain the nucleus are functionally closer to phototrophs [1]. In addition, in *M. rubrum*, the ingested nucleus of the cryptophyte prey is required to enlarge, divide, and sustain the function of kleptoplasts [18]. It has been shown that the ingested nucleus is transcriptionally active in the ciliate cell and its transcriptome changes after ingestion. Such kleptoplast enlargements have not been observed in kleptoplastic organisms that do not retain the nucleus of ingested algae [19]. However, other than in *M. rubrum*, the function of the ingested nuclei has not yet been studied; thus, it remains unclear whether commonalities exist in the function of ingested nuclei and the evolutionary relationship between kleptoplasty and secondary/tertiary endosymbioses.

Among several kleptoplastic organisms, we have studied kleptoplasty in the dinoflagellates *Nusuttodinium* spp., which transiently retain kleptoplasts derived from a cryptomonad prey [6, 19–23]. We previously showed that *Nusuttodinium poecilochroum* digests the cryptomonad nucleus within a few hours of ingestion, while keeping but not enlarging the kleptoplast for about a week. In contrast, *Nusuttodinium aeruginosum* retains the cryptomonad nucleus, enlarges the kleptoplast more than 20-fold, and keeps it for more than a month after ingestion [19, 20]. In *N. aeruginosum*, the ingested cryptomonad nucleus is inherited by only one of the two daughter cells during cell division. The cell without the cryptomonad nucleus stops enlarging the kleptoplast and, after three rounds of cell division, starts to digest it [20]. These observations suggest that the retention of the cryptomonad nucleus is required to enlarge the kleptoplast and maintain its function for a longer period. We also found an obligate heterotrophic species feeding on a cryptomonad that is closely related to *Nusuttodinium* spp. [24]. Taken together, *Nusuttodinium* spp. are ideal organisms to study the evolution of kleptoplasty.

In order to obtain insights into how the ingested cryptomonad nucleus supports the growth and function of the kleptoplast, we examined the changes in the structure and transcriptome of the nucleus during the course of kleptoplasty in *N. aeruginosum*. In addition, we compared the effects of high light stress on *N. aeruginosum* in cells with and without the cryptomonad nucleus. Here we show that similar to *M. rubrum*, cryptomonad genes involved in metabolism and translation are upregulated after being ingested by *N. aeruginosum*, which is in accordance with polyploidization of the nucleus. Furthermore, transcriptional responses to illumination are almost lost in the cryptomonad nucleus after ingestion. Moreover, our results suggest that the cryptomonad nucleus reduces high light damage on the kleptoplast and *N. aeruginosum*. Because polyploidization and loss of transcriptional regulation were also observed in nucleomorph and organelle genomes, these events are common trends in evolution of kleptoplasty and the course of chloroplast establishment by secondary/tertiary endosymbioses.

## Materials and methods

### Cell cultures

The dinoflagellate *N. aeruginosum* and the cryptophytes *Chroomonas* spp. Dc01 and HrL01 were collected in previous studies [19–21]. To remove bacteria in respective cultures, the cells were washed several times with sterilized AF-6 medium (an inorganic medium; https://mcc.nies.go.jp/medium/en/af6.pdf) by micropipetting. The cells were incubated at 20 °C under continuous light (10 µmol photons m^−2^ s^−1^) and, unless otherwise indicated, in 25-cm^2^ tissue culture flasks without agitation. *N. aeruginosum* was fed *Chroomonas* sp. Dc01 on a regular basis.

### RNA preparation for transcriptomic analyses

To analyze the transcriptome of *N. aeruginosum* in which the nuclei and kleptoplasts derived from *Chroomonas* sp. Dc01 were being enlarged, free *Chroomonas* sp. were removed from the coculture using a plankton-net with 5-µm pores (NY5-HC, SEFER) to inhibit *N. aeruginosum* newly ingesting *Chroomonas* sp. Then, *N. aeruginosum* with kleptoplasts (35.2 × 10^2^ ± 1.70 cells/mL; three independent cultures) were inoculated into 175 mL of fresh AF-6 medium in a 75-cm^2^ cell culture flask. The culture was incubated with illumination for 4 days so that the kleptoplasts were enlarged. After being incubated in the dark for 24 h (hour 0), the culture was illuminated for 12 h and then returned to the dark. For comparison, free-living *Chroomonas* sp. Dc01 (111.9 × 10^3^ ± 7.23 cells/mL; three independent cultures) were cultured alone under the same conditions.

Cells were collected at hours 0 (just before illumination), 1, 6 (during the light period), 12 (just before dark), and 13 (1 h after the transfer to dark) by centrifugation at 1500 × *g* for 5 min. The cell pellet was immediately frozen in liquid nitrogen. Total RNA was extracted using a NucleoSpin RNA XS kit (TaKaRa). To construct a cDNA library of 250–300 bp, 0.7–2.7 μg (*N. aeruginosum* culture), and 5.2–9.4 μg (free-living *Chroomonas* sp. Dc01 culture) of total RNA were used. Paired-end sequencing was performed using the Illumina sequencing platform (Novaseq 6000) according to the manufacturer’s instructions (Illumina).

### *De novo* assembly and annotation of *Chroomonas* sp. RNA-seq data

The RNA-seq raw reads were cleaned up using cutadapt ver. 1.81 [25] by trimming low-quality ends (<QV30) and adapter sequences and by discarding reads shorter than 50 bp. The trimmed reads of the free *Chroomonas* sp. culture were assembled *de novo* using Trinity ver. 2.0.6 [26] with the paired-end mode and the option “--min_contig_length 300,” and the output fasta files were clustered together using CD-HIT ver. 4.6.8 [27] with the “-c 0.95” option. When splicing variants of a gene were found, the longest transcript was selected as a representative mRNA sequence. Open reading frames (*orf*s) were predicted by TransDecoder ver. 5.1.0 (http://transdecoder.github.io). The contigs were used as the reference for the *Chroomonas* sp. Dc01 transcripts. To obtain gene expression scores, one side of the trimmed paired-end reads was mapped to the reference by Bowtie2 ver. 2.3.4.1 [28]. SAMtools ver. 1.8 [29], BEDtools ver. 2.19.1 [30], and R ver. 3.5.3 [31] were used to calculate the number of reads mapped to the contigs (raw count).

### Analyses of transcriptomic changes

To compare the nuclear transcriptome of free-living *Chroomonas* sp. Dc01 with that of *Chroomonas* sp. Dc01 ingested by *N. aeruginosum*, mapped read counts (count per million: CPM) of each contig at five time points were averaged. The genes with low mapped read counts (average CPM < 1) were omitted. The data were then normalized between free-living and ingested *Chroomonas* sp., and differentially expressed genes (DEGs) were identified by edgeR ver. 3.24.3 [32] in R. Contigs of ingested *Chroomonas* sp. were defined as DEGs when the false discovery rate (FDR) was <0.01 and the absolute value of logFC (log2-fold-change of ingested state vs. free-living state) was >2. Upregulated and downregulated genes were categorized without redundancy according to the second level terms of the Kyoto Encyclopedia of Genes and Genomes (KEGG) [33]. The KEGG Orthology ID assignment was performed for all *orf*s in the KAAS pipeline [34].

To compare changes in the transcriptome in light and dark conditions, the count data were analyzed by TCC ver. 1.22.1 [35] in R. The low count contigs (CPM < 1) were omitted. Principal component analysis (PCA) of count data, which were normalized by TCC, was performed using R. DEGs of free-living *Chroomonas* sp. were confirmed only when FDR was <0.01 and the absolute value of logFC (log2 fold-change of either hour 1, 16, 12, or 13 vs. hour 0) was >2. To compare expression patterns between free-living and ingested *Chroomonas* sp., the count data of both were normalized together by TCC in R. The logFC (log2 fold-change against the value of free-living *Chroomonas* sp. at hour 0) was calculated based on the normalized count data + 1. The logFC values of 2708 DEGs were clustered by the *k*-means method (*k* = 7).

### Quantification of SYBR green fluorescence and chloroplast fluorescence

Free *Chroomonas* sp. was removed from the coculture, and *N. aeruginosum* were starved in the light for 14 days until they had digested most of the kleptoplasts derived from *Chroomonas* sp. Then, the starved *N. aeruginosum* were fed *Chroomonas* sp. (the ratio of *N. aeruginosum*:*Chroomonas* sp. was 1:3) for 9 h until the majority of the cells had ingested *Chroomonas* sp. in the light. After free *Chroomonas* sp. was removed, *N. aeruginosum* with kleptoplasts (4.0 × 10^2^ cells/mL) were cultured in 175 mL of AF-6 medium in 75-cm^2^ cell culture flasks in the light. One milliliter of the culture was fixed with 0.3% glutaraldehyde, stained with 1/10,000-diluted SYBR Green I (LONZA) for 1 h, and observed using fluorescence microscopy. The intensities of kleptoplast red fluorescence and SYBR Green fluorescence of *Chroomonas* sp. nuclei were quantified with ImageJ [36].

### Quantitative PCR (qPCR)

After starvation of *N. aeruginosum*, it was fed *Chroomonas* sp. HrL01 in the light instead of Dc01 to avoid amplification of residual *Chroomonas* sp. Dc01 DNA by qPCR. Free *Chroomonas* sp. HrL01 were removed, and *N. aeruginosum* were starved for 5 days in the light until they had digested most of the kleptoplasts derived from *Chroomonas* sp. HrL01. The starved *N. aeruginosum* were then fed *Chroomonas* sp. Dc01 (the ratio of *N. aeruginosum*:*Chroomonas* sp. was 1:2) for 9 h until the majority of cells had ingested *Chroomonas* sp. Free *Chroomonas* sp. were removed, and the *N. aeruginosum* with kleptoplasts were inoculated into 240 mL of fresh AF-6 medium in 75-cm^2^ cell culture flasks (3.33 × 10^2^ ± 0.577 cells/mL; three independent cultures). Subsequently, on day 0, the *N. aeruginosum* with kleptoplasts were cultured in the light.

Total DNA was extracted with a DNeasy^®^ Plant Mini Kit (QIAGEN). qPCR was performed with 70 pg of total DNA for each sample using a StepOne-Plus Real-Time PCR system (Life Technologies) and Power SYBR Green Master Mix (Life Technologies). The values of the respective genes were normalized with that of *N. aeruginosum* 18S rDNA. The primer sequences are shown in Supplementary Table 3.

### Quantitative reverse transcription-PCR (qRT-PCR)

The *N. aeruginosum* cells were starved, as described in the “Quantification of SYBR Green fluorescence…” section, and then *Chroomonas* sp. cells were added to the culture (the ratio of *N. aeruginosum*:*Chroomonas* sp. cells was 1:10). After the majority of *N. aeruginosum* cells had ingested *Chroomonas* sp. (9 h after the addition of *Chroomonas* sp.), free *Chroomonas* sp. cells were removed (day 0), and then *N. aeruginosum* with kleptoplasts (7.0 × 10^2^ cells/mL) were cultured in 175 mL of AF-6 medium in 75-cm^2^ cell culture flasks in the light. *N. aeruginosum* cells were harvested from 50 mL of culture at days 0, 6, and 12, and the cell pellets were stored at −80 °C. To quantify mRNA levels of free *Chroomonas* sp., 50 mL of culture was harvested just after the addition of *Chroomonas* sp. to the starved *N. aeruginosum*, and the cell pellets were stored at −80 °C. Immediately before RNA extraction, 2 mL of *Cyanidioschyzon merolae* 10D culture (OD_750_ = 0.406), which was exponentially growing in continuous light [37], was added to each sample as an internal control (i.e., equivalent to the culture volume).

Total RNA was extracted using a NucleoSpin RNA XS kit (TaKaRa). cDNA was synthesized from 500 ng of total RNA using a mixture of a random hexamer and oligo dT primer (1:9 ratio in concentration) with PrimeScript RTase (TaKaRa). qPCR analyses were performed, as described above. The values of the respective genes were normalized with the values of *C*. *merolae DRP3* mRNA and the number of *Chroomonas* sp. nuclei per culture volume. The primer sequences are shown in Supplementary Table 3.

### Cultures at different light intensities

To examine the cellular growth of *N. aeruginosum* with kleptoplasts, free *Chroomonas* sp. Dc01 was removed from the coculture, and *N. aeruginosum* was starved for 12 days in the light until 40% of the cells had lost the nuclei but not the kleptoplasts derived from *Chroomonas* sp. Then, *N. aeruginosum* (23.5 × 10^2^ ± 1.55; three independent cultures) and free-living *Chroomonas* sp. Dc01 (69.3 × 10^3^ ± 2.52 cells/mL; three independent cultures; chlorophyll *a* concentration, which was determined as described by Jeffrey and Humphrey [38], was equivalent to that of the *N. aeruginosum* culture) were inoculated respectively into 6 mL of fresh AF-6 medium in a 6-well microplate and cultured in the dark or in the light (10, 50, 100, or 200 µmol photons m^−2^ s^−1^) for 5 days.

## Results

### Establishment of an axenic coculture of the dinoflagellate *N. aeruginosum* and the cryptomonad *Chroomonas* sp.

**Fig. 1:**
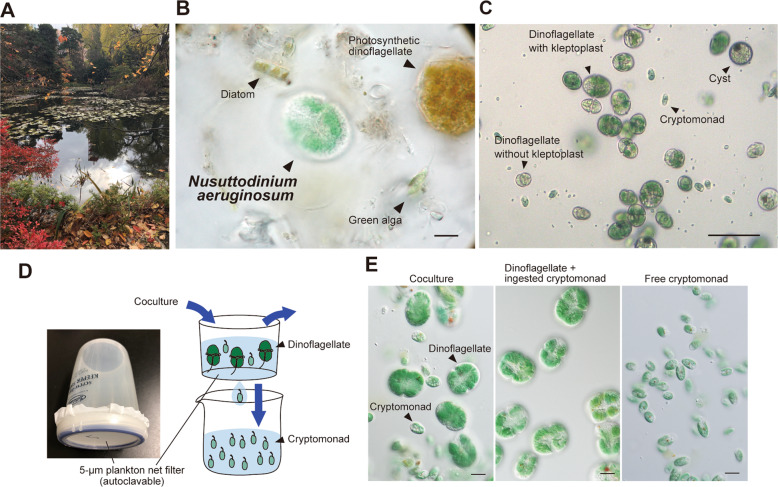
Habitat and establishment of the axenic coculture of the dinoflagellate *N. aeruginosum* and the cryptomonad *Chroomonas* sp. Dc01. **a** The pond in Hokkaido prefecture, Japan from which *N. aeruginosum* and *Chroomonas* sp. Dc01 were collected. **b** Micrograph of a water sample from the pond showing the coexistence of *N. aeruginosum* and unicellular algae. The cells were collected on September 24, 2010 [19]. Scale bar = 10 μm. **c** Micrograph showing the axenic coculture of *N. aeruginosum* and *Chroomonas* sp. The coculture contains *Chroomonas* sp. as a source kleptoplast of *N. aeruginosum* as well as *N. aeruginosum* cells with or without kleptoplast, and cysts. Scale bar = 50 μm. **d** Photograph of a plankton-net filter and a schematic drawing showing the separation of *N. aeruginosum* cells with *Chroomonas* sp.-derived kleptoplasts from free *Chroomonas* sp. (those had not been ingested by *N. aeruginosum*). A 5-µm diameter plankton-net filter was attached to the bottom of the sieve. The coculture of *N. aeruginosum* and *Chroomonas* sp. was filtrated to separate free *Chroomonas* sp. from *N. aeruginosum* with kleptoplasts. *N. aeruginosum* cells on the filter were washed several times before further experiments. **e** Micrographs showing the coculture of *N. aeruginosum* cells with *Chroomonas* sp.-derived kleptoplasts and free *Chroomonas* sp. (left), filtered *N. aeruginosum* cells that are separated from free *Chroomonas* sp. (middle), and free *Chroomonas* sp. in the flow-through fraction. Scale bar = 10 μm.

In our previous study, we isolated the kleptoplastic dinoflagellate *N. aeruginosum* and its prey, *Chroomonas* sp. Dc01 from a mixture of unicellular organisms in the same freshwater pond (Fig. 1a, b); however, we had not yet established their axenic cultures [19]. Within the scope of our testing to date, *N. aeruginosum* ingests *Chroomonas* spp. Dc01 and HrL01 but not other cryptophytes or eukaryotic algae. In the present study, to avoid the possible effects of contaminated bacteria on the activities of the dinoflagellate and cryptomonads, we first removed bacteria from the cultures of *N. aeruginosum* and *Chroomonas* spp. by serial micropipetting and transferring the cells to a sterilized inorganic medium under a microscope. The strains were maintained under continuous light conditions (10 µmol photons m^−2^ s^−1^) without agitation at 20 °C. *N. aeruginosum* cells were fed *Chroomonas* sp. Dc01 (Fig. 1c). For 3 years after the sterilization, we have confirmed that the respective cultures are axenic by phase-contrast and differential interference contrast microscopy with a 100× objective lens and based on no colony formation on Luria Broth agar plate (9 cm in diameter) 3 days after streaking 0.5 mL of cocultured medium and incubation at 20 °C for 7 days.

To compare *Chroomonas* sp. in the free-living population with those that were ingested by *N. aeruginosum*, we developed a filtration procedure to fractionate *Chroomonas* sp. that had and had not been ingested by *N. aeruginosum*. When the coculture of *N. aeruginosum* and *Chroomonas* sp. Dc01 was passed through a filter with 5-µm pores (Fig. 1d), all *N. aeruginosum* cells remained on the filter, while all free *Chroomonas* sp. passed through (Fig. 1e).

### Genes involved in metabolism, protein synthesis, and DNA replication are upregulated in the cryptomonad nucleus after ingestion by the dinoflagellate

We previously observed that during cell division of *N. aeruginosum*, the ingested nucleus was inherited by only one of the two daughter cells. The daughter cell with the *Chroomonas* sp. nucleus continued to enlarge the kleptoplast while that without the nucleus did not. These results suggested that the ingested nucleus is required for kleptoplast enlargement and that the ingested nucleus is probably transcriptionally active [19, 20].

**Fig. 2:**
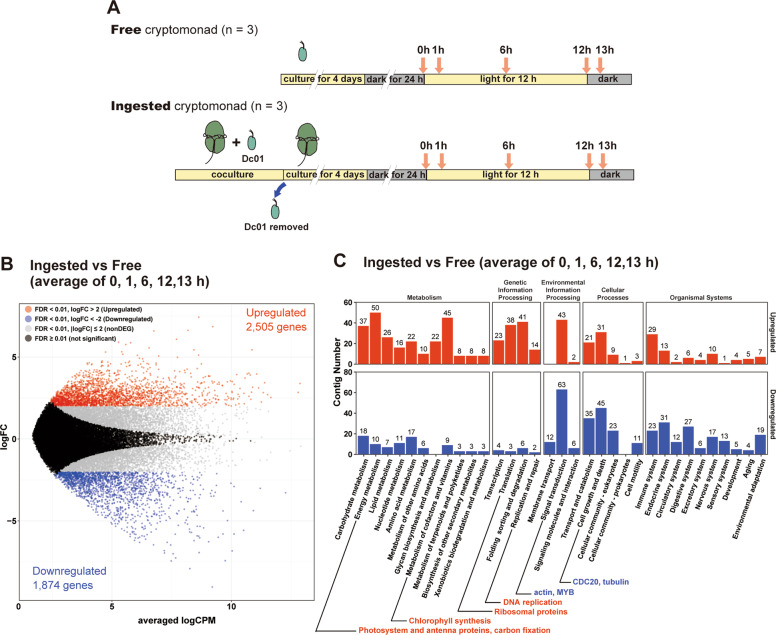
Comparison of the nuclear transcriptome between *Chroomonas* sp. cells before and after ingestion by the dinoflagellate *N. aeruginosum*. **a** Schematic diagrams showing the culture of free-living *Chroomonas* sp. Dc01 and that of *Chroomonas* sp. Dc01 ingested by *N. aeruginosum* used for comparison of the nuclear transcriptomes of *Chroomonas* sp. Dc01 in each state. Both cultures were pre-incubated in the dark for 24 h. Then, from hour 0, the cultures were subjected to 12-h-light and subsequently dark. Nuclear transcriptomes of *Chroomonas* sp. Dc01 were examined at hours 0 (just before illumination), 1, 6 (during the light period), 12 (just before the dark), and 13 (during the dark period). To inhibit *N. aeruginosum* from ingesting any new *Chroomonas* sp. Dc01, free-living *Chroomonas* sp. were removed from the coculture 4 days prior to the dark preincubation. **b** M-A plot showing 2505 upregulated and 1874 downregulated contigs from *Chroomonas* sp. ingested by *N. aeruginosum* cells and free-living *Chroomonas* sp. cells. The averages of respective mRNA levels at five time points (hours 0, 1, 6, 12, and 13) were compared. Upregulated and downregulated contigs were defined as those that had FDR < 0.01 and logFC > 2 (indicated in red) and FDR < 0.01 and logFC < −2 (indicated in blue), respectively (three independent cultures). The *x*-axis represents averaged logCPM, and the y-axis represents the base 2 logarithm of the fold change to the free-living condition. **c** Comparison of the number of upregulated and downregulated contigs whose functions were assigned to respective KEGG functional categories.

To examine how the transcriptome changes upon ingestion by *N. aeruginosum*, we compared the nuclear transcriptome of free (free-living *Chroomonas* sp.) and ingested *Chroomonas* sp. (5 days after ingestion by *N. aeruginosum*) by RNA-seq analysis (Fig. 2a). To this end, cells were precultured in the dark for 24 h, illuminated for 12 h, and again returned to the dark. Mapped read counts (count per million: CPM) of each contig at five time points [hour 0 (just before illumination), 1, 6, 12 (during illumination), and 13 (1 h after the transfer to the dark)] were averaged and then compared (Fig. 2b, c). From a total of 70,969 *Chroomonas* sp. nuclear contigs, 2505 (3.53%) and 1874 (2.64%) contigs were upregulated (FDR < 0.01, logFC of ingested state vs free-living state > 2) and downregulated (FDR < 0.01, logFC of ingested state vs free-living state < −2), respectively, after ingestion (Fig. 2b; Supplementary Table 1). To further evaluate the functional change of the *Chroomonas* sp. nucleus following ingestion, the upregulated and downregulated contigs were assigned to the KEGG database [33] (Fig. 2c). Upregulated contigs were predominantly found in “Metabolism” and “Genetic information processing” categories (Fig. 2c). These included contigs annotated as genes encoding components of photosystems, proteins involved in carbon fixation (e.g., carbonic anhydrase and proteins involved in carbohydrate metabolism), proteins involved in translation (e.g., ribosomal proteins), and proteins involved in nuclear DNA replication (MCM proteins and DNA primase) (Fig. 2c; Supplementary Table 1). In addition, these contigs included genes encoding superoxide dismutase, ascorbate peroxidase, and thioredoxin reductase, which are involved in the dissipation of reactive oxygen species (ROS) [39–41] (Fig. 2c; Supplementary Table 1). In contrast, “Environmental information processing,” “Cellular Processes,” and “Organismal systems” categories predominantly contained downregulated contigs (Fig. 2c). Contigs annotated as genes encoding actin, tubulin, CDC20 [42], and CDC14 [43], which are involved in M-phase progression, were also downregulated.

### The cryptophyte nucleus almost loses its transcriptional response to dark–light shift after ingestion by the dinoflagellate

**Fig. 3:**
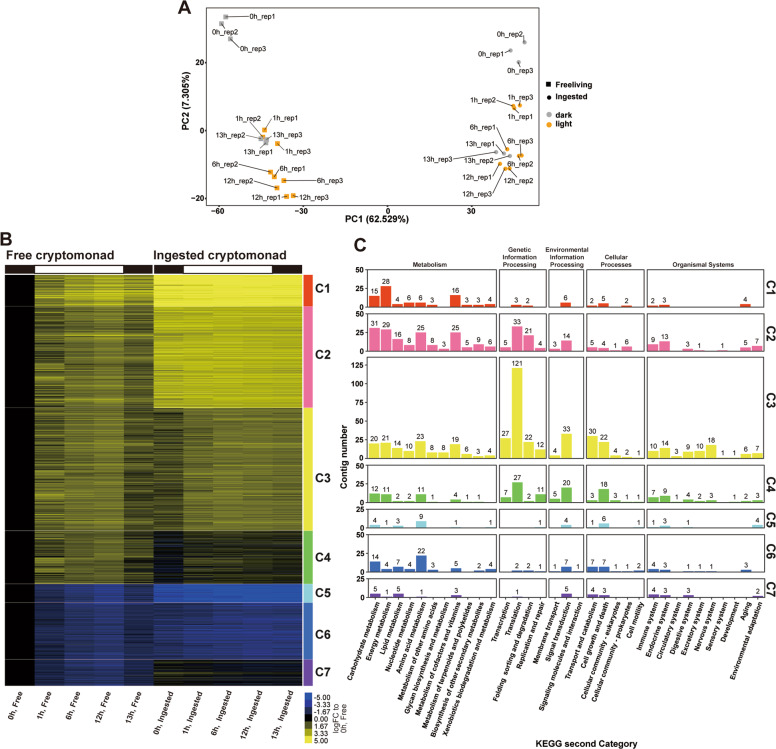
Comparison of the nuclear transcriptomic change following illumination in *Chroomonas* sp. cells before and after ingestion by the dinoflagellate *N. aeruginosum*. The RNA-seq data were obtained from the process described in Fig. 2a. **a** PCA of the transcriptomic changes of the free and ingested *Chroomonas* sp. following illumination. The raw count data were normalized with TCC using R and then plotted (low count contigs were omitted before normalization). The square and circle correspond to free-living and ingested *Chroomonas* sp. at the indicated time point, respectively. The color represents the presence (orange) or absence (gray) of light illumination. **b** Heatmap showing the expression patterns of DEGs (log2-fold-change at any time point from hour 0 > 2; FDR < 0.01; three independent cultures) in the free-living *Chroomonas* sp. and a comparison with those in the ingested *Chroomonas* sp. The levels in the free-living *Chroomonas* sp. at hour 0 were defined as “0.” Yellow and blue indicate upregulation and downregulation of the gene, respectively, compared with the level at hour 0 in the free-living *Chroomonas* sp. The clustering was performed by the *k*-means method. **c** Comparison of the number of contigs whose functions were assigned to respective KEGG functional categories of second level terms in respective clusters. Colors correspond to clusters 1–7 in (**b**).

As described later, both enlargements of the kleptoplast and *N. aeruginosum* growth depend on light (i.e., photosynthesis by the kleptoplast). In addition, it is well known that illumination largely changes transcriptome in photosynthetic organisms e.g. [44, 45]. To assess how the ingested *Chroomonas* sp. nucleus responds to dark/light shift, we examined and compared the transcriptomic changes of free and ingested *Chroomonas* sp. (5 days after ingestion by *N. aeruginosum*) following illumination after preculture in the dark. Cells were precultured in the dark for 24 h, illuminated for 12 h, and again returned to the dark. The nuclear transcriptome of free and ingested *Chroomonas* sp. was examined at hours 0 (just before illumination), 1, 6, 12 (during illumination), and 13 (1 h after the transfer to the dark) (Figs. 2a and 3).

PCA showed that a conspicuous difference existed between the free-living and ingested *Chroomonas* sp. along the PC1 axis (Fig. 3a). Along the PC2 axis, the transcriptomes in free-living states were separated according to the dark (hour 0: just before illumination) and light (hours 1, 6, and 12) conditions. The hour 13 (1 h after the dark shift) transcriptomes moved toward those of hour 0 in both the free-living and ingested states (Fig. 3a). Furthermore, the transcriptomes at any time point were largely different between the free-living and ingested states (Fig. 3a).

In the PCA analysis, there was less difference upon the illumination (PC2 axis) in the ingested state than in the free-living state (Fig. 3a; differences in principal component 2 score between hour 0 and 1 and between hour 0 and 12 in the ingested state were significantly less than those in free-living state; *p* = 0.0044 and 0.0046, respectively, *t*-test), which suggests that the transcriptomic response of the *Chroomonas* sp. nucleus to light was compromised after it was ingested by *N. aeruginosum*. To further investigate this finding, we identified all DEGs following illumination in the free-living state (FDR < 0.01 and |logFC of either hour 1, 16, 12, or 13 vs. hour 0 | > 2; 2708 contigs, Supplementary Table 2) and then compared their expression patterns with those in the ingested state (Fig. 3b). As suggested by the PCA analysis, changes in levels of mRNAs that were identified as DEGs in the free-living state were abolished or compromised in the ingested state (Fig. 3b). By *k*-means clustering, the DEGs in the free-living state were classified according to the extent by which the magnitude of the changes was reduced in the ingested state (Fig. 3b). Relative to the free-living state, clusters 1 (208 contigs) and 2 (668 contigs) were highly expressed in the ingested state regardless of dark or light conditions. In these clusters, the majority of the contigs were assigned to the “metabolism” category by KEGG classification. Clusters 3 (812 contigs) and 4 (350 contigs) were slightly upregulated in the ingested state following illumination, and they included a majority of “translation” genes. Relative to the free-living state, clusters 5 (122 contigs) and 6 (375 contigs) were downregulated in the ingested state regardless of dark or light conditions. In the ingested state, Cluster 7 (173 contigs) almost lost the change of the DEGs in the free-living state following illumination (Fig. 3b, c, Supplementary Table 2).

In summary, the analyses of transcriptomes showed that the *Chroomonas* sp. nucleus almost lost its transcriptional response to illumination following ingestion by *N. aeruginosum*. Furthermore, contigs annotated as genes involved in metabolism were upregulated in the ingested state relative to the free-living state regardless of light conditions, while contigs annotated as genes involved in translation were slightly upregulated following illumination although the magnitude of the changes was reduced compared with the free-living state.

### Increase in the ploidy of the cryptomonad genome and mRNAs after ingestion by the dinoflagellate

**Fig. 4:**
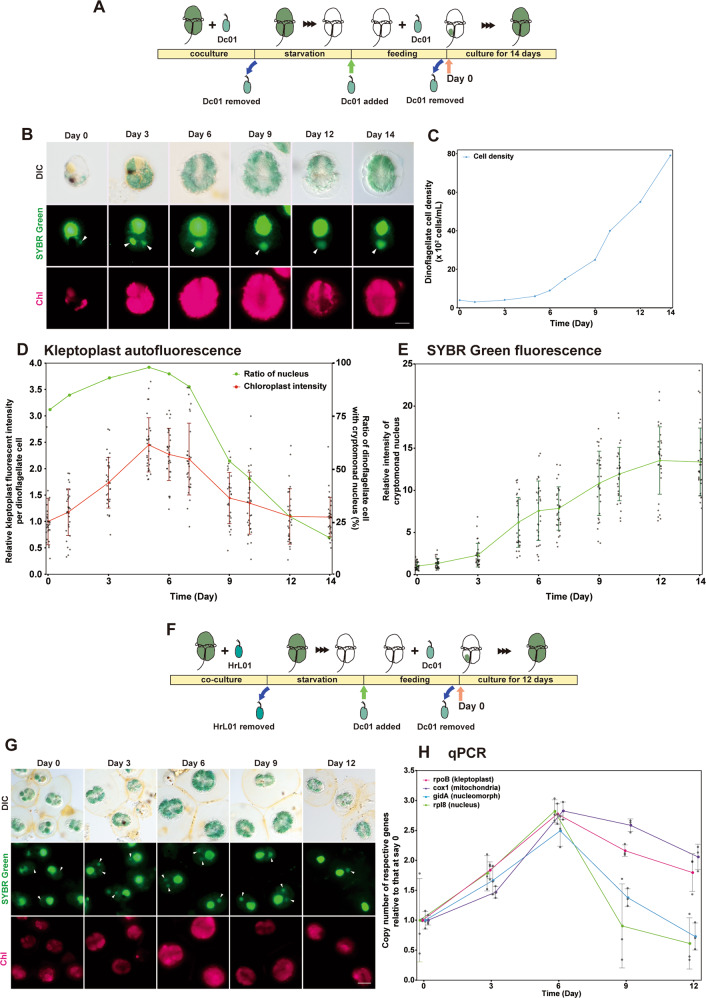
Change in the ploidy levels of the nucleus, mitochondrion, nucleomorph, and chloroplast of *Chroomonas* sp. after ingestion by *N. aeruginosum*. **a** Schematic diagrams showing the coculture of *N. aeruginosum* and *Chroomonas* sp. Dc01 used to investigate the change in the ploidy level of *Chroomonas* sp. Dc01 ingested by *N. aeruginosum* in (**b**–**e**). *Chroomonas* sp. Dc01 was removed from the coculture for *N. aeruginosum* cells to digest most of the kleptoplasts derived from *Chroomonas* sp. (~95 and 60% of *N. aeruginosum* cells had completely digested the nucleus and kleptoplast derived from *Chroomonas* sp., respectively). Then, the starved *N. aeruginosum* cells were again fed *Chroomonas* sp. until approximately 75% of the cells had ingested *Chroomonas* sp. After this, free-living *Chroomonas* sp. was removed from the coculture (day 0) and *N. aeruginosum* cells with kleptoplasts were cultured for 14 days under continuous light (10 µmol photons m^−2^ s^−1^). **b** Micrographs showing the change in the nucleus and kleptoplast derived from *Chroomonas* sp. in *N. aeruginosum* cells. Cells were stained with SYBR Green. Images of differential interference contrast (DIC), SYBR Green staining, and kleptoplast red fluorescence (Chl) are shown. The arrowhead indicates a nucleus derived from *Chroomonas* sp. The cell in the image from day 3 ingested two nuclei. Scale bar = 10 μm. **c** Change in *N. aeruginosum* cell density. **d** Change in the ratio of *N. aeruginosum* cells retaining the nucleus derived from *Chroomonas* sp. and kleptoplast fluorescence intensity per *N. aeruginosum* cell. For kleptoplast fluorescence, the average intensity at day 0 was defined as “1.0.” The kleptoplast fluorescence intensity was quantified both in *N. aeruginosum* cells with and without the nucleus derived from *Chroomonas* sp. The error bars represent standard deviation (*n* = 30 cells for each time point). **e** Change in SYBR Green fluorescence intensity per *Chroomonas* sp. nucleus ingested by *N. aeruginosum*. The average at day 0 was defined as “1.0.” The error bars represent standard deviation (*n* = 30 nuclei). **f** Schematic diagrams showing the coculture of *N. aeruginosum* and *Chroomonas* sp. used to investigate the change in the ploidy level of *Chroomonas* sp. Dc01 ingested by *N. aeruginosum* in (**g**, **h**). To avoid qPCR amplification of residual DNA from *Chroomonas* sp. Dc01 after starvation of *N. aeruginosum, Chroomonas* sp. HrL01 instead of Dc01 was fed to *N. aeruginosum* before starvation and then Dc01 was added to the culture. Others are the same as in (**a**). **g** Micrographs showing that described in (**b**) except that they show the cells from (**f**) instead of (**a**). **h** qPCR showing changes in ploidy of nuclear (*rpl8* gene), mitochondrial (*cox1* gene), nucleomorph (*gidA* gene), and kleptoplast (*rpoB* gene) genomes of *Chroomonas* sp. Dc01 ingested by *N. aeruginosum*. The values were normalized with values of *N. aeruginosum* 18S rDNA. The average at day 0 was defined as “1.0” for respective genomes. The error bars represent standard deviation (three independent cultures).

The transcriptome analyses showed that genes involved in nuclear DNA replication (S phase) were upregulated while certain M-phase genes were downregulated in *Chroomonas* sp. after ingestion by *N. aeruginosum*. Thus, we hypothesized that the *Chroomonas* sp. nucleus continued to replicate in *N. aeruginosum*. To test this prediction, we examined the change in the size of the nucleus and the ploidy of nuclear and organellar genomes of *Chroomonas* sp. after ingestion by *N. aeruginosum*. In addition, we examined the relationship between the change in the nucleus and kleptoplast after ingestion after removing free *Chroomonas* sp. (Fig. 4).

The size and fluorescence intensity of kleptoplasts per *N. aeruginosum* cell peaked at 5–6 days and then decreased gradually (Fig. 4b, c; note that some *N. aeruginosum* ingested two or more *Chroomonas* sp. cells during feeding in Fig. 4a). After removal of free *Chroomonas* sp. (day 0), the percentage of *N. aeruginosum* with *Chroomonas* sp. nuclei increased until day 5 because of the death of cells that had not ingested *Chroomonas* sp. From then on, the percentage decreased as a result of unequal inheritance of the *Chroomonas* sp. nuclei by *N. aeruginosum* daughter cells during cell division [20], which coincided with the reduction of kleptoplast size per *N. aeruginosum* cell on average (Fig. 4a–d).

The size and fluorescent intensity of SYBR Green staining of the ingested nucleus continued to increase by up to ten times the original size before reaching a plateau at day 12 (Fig. 4b, e). qPCR analysis also showed that the copy number of *Chroomonas* sp. Dc01 nuclear *rpl8* gene (per *N. aeruginosum* nuclear rDNA gene) increased until day 6 and then decreased (Fig. 4f–h). A discrepancy between the change in nuclear size and SYBR Green intensity (Fig. 4e), which kept increasing, and the qPCR result, in which the copy number per *N. aeruginosum* nuclear genome decreased from day 6 (Fig. 4h), was probably due to the ratio of *N. aeruginosum* cells with *Chroomonas* sp. nuclei, which decreased from day 6 because of the unequal inheritance of nuclei during *N. aeruginosum* cell division (Fig. 4d, g). In addition to the nuclear genome, qPCR showed that DNA content of mitochondrial (*cox1*), nucleomorph (*gidA*), and chloroplast (*rpoB*) genomes of *Chroomonas* sp. Dc01 also increased until day 6 and then decreased.

**Fig. 5:**
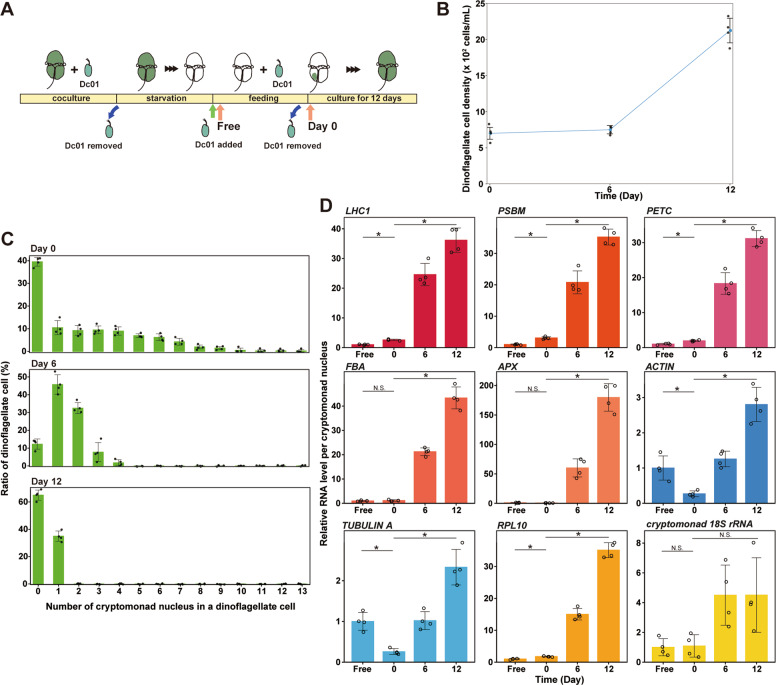
Change in the mRNA and rRNA levels of the nucleus of *Chroomonas* sp. after ingestion by *N. aeruginosum*. **a** Schematic diagrams showing the coculture of *N. aeruginosum* and *Chroomonas* sp. Dc01 used to investigate the change in mRNA and rRNA levels of *Chroomonas* sp. ingested by *N. aeruginosum* in (**b**–**d**). *Chroomonas* sp. Dc01 was removed from the coculture for *N. aeruginosum* cells to digest most of the kleptoplasts derived from *Chroomonas* sp. (~95% and 60% of *N. aeruginosum* cells had completely digested the nucleus and kleptoplast derived from *Chroomonas* sp., respectively). The starved *N. aeruginosum* cells were again fed *Chroomonas* sp. until ~60% of the cells had ingested *Chroomonas* sp. Then, the free-living *Chroomonas* sp. was removed from the coculture (day 0) and *N. aeruginosum* cells with kleptoplasts were cultured for 12 days under continuous light (10 µmol photons m^−2^ s^−1^). **b** Change in *N. aeruginosum* cell number after the removal of free *Chroomonas* sp. **c** Histogram showing the change in the ratio of *N. aeruginosum* cells possessing cryptomonad nuclei and the number of the nuclei per cell. **d** RT-qPCR showing the changes in mRNA levels per *Chroomonas* sp. nucleus of *LHC1* (*LIGHT HARVESTING COMPLEX 1*), *PSBM*, *PETC, FBA, APX, RPL10, ACTIN*, and *TUBULIN A* mRNAs, and 18S rRNA, upon and after ingestion. The values were normalized with that of an internal control, which was equivalent to the culture volume, and the number of *Chroomonas* sp. nuclei per culture volume. The averaged value of free-living *Chroomonas* sp. was defined as “1.0.” The levels in the free-living *Chroomonas* sp. were determined in the culture immediately after the addition of *Chroomonas* sp. to the starved *N. aeruginosum* culture. The error bars represent standard deviation (four independent cultures). *P < 0.05; *N.S*., no significant difference (*t*-test).

Transcriptome analyses showed that many contigs annotated as *Chroomonas* sp. nuclear genes, especially those involved in metabolism and translation, were upregulated after ingestion. However, the upregulation or downregulation of a particular gene in RNA-seq results indicates a respective increase or decrease in the ratio of the mRNA levels of the gene to the total mRNA of *Chroomonas* sp. Thus, upregulation or downregulation does not necessarily indicate a respective increase or decrease in the absolute level of mRNA (i.e., the mRNA level of a given gene per *Chroomonas* sp. nucleus). To investigate the relationship between the upregulated and downregulated genes and the increase in the ploidy of the *Chroomonas* sp. nucleus, we examined the change in mRNA levels of some genes and that of 18S rRNA (to assess the relative number of ribosomes) per *Chroomonas* sp. nucleus in the course of ingestion and polyploidization (Fig. 5). To this end, the starved *N. aeruginosum* cells were fed *Chroomonas* sp. for 9 h and, afterwards, free *Chroomonas* sp. cells were removed from the culture (day 0) before *N. aeruginosum* with kleptoplasts were cultured for 12 days (Fig. 5a). By the removal of free *Chroomonas* sp., ~60% of the *N. aeruginosum* cells ingested 1–14 *Chroomonas* sp. cells. Subsequently, *N. aeruginosum* cells that did not ingest *Chroomonas* sp. started to die, resulting in a decrease in the ratio of *N. aeruginosum* cells without *Chroomonas* sp. nuclei at day 6 (Fig. 5b, c). After the removal of free *Chroomonas* sp. from the culture at day 0, the number of *Chroomonas* sp. nuclei per *N. aeruginosum* cell also decreased because of the lack of nuclear division of *Chroomonas* sp. in *N. aeruginosum* cells and the unequal inheritance during *N. aeruginosum* cell division (Fig. 5b, c).

qRT-PCR showed that the 18S rRNA level per *Chroomonas* sp. nucleus remained almost constant upon ingestion and then increased in the *N. aeruginosum* cell. Of six genes that were found to be upregulated DEGs in the transcriptome analysis, mRNA levels of *PSBM*, *PETC*, *LHC1*, and *RPL10* per *Chroomonas* sp. nucleus increased upon ingestion (i.e., free vs. hour 0 in Fig. 5d). After ingestion, mRNA levels of all the six genes continued increasing (Fig. 5d) in accordance with polyploidization of the ingested nucleus (Fig. 4). In contrast, mRNA levels of the downregulated DEGs *ACTIN* and *TUBULIN A* per *Chroomonas* sp. nucleus decreased upon ingestion and then increased afterward, although the magnitudes of their increase were smaller than those of *PSBM*, *PETC*, *LHC1 FBA, APX*, and *RPL10* (Fig. 5d). Therefore, the increase or decrease of mRNA levels upon ingestion and the differential magnitudes of the increase during polyploidization of the ingested nucleus probably contributed to the upregulation or downregulation of genes in the transcriptome (Fig. 2).

### The ingested cryptophyte nucleus allays high light-induced damage to the kleptoplasts and dinoflagellate cell

Above results suggest that the ingested nucleus supports the growth of the kleptoplast in *N. aeruginosum* cells. To elucidate any additional effects of the ingested *Chroomonas* sp. nucleus on the kleptoplast and/or dinoflagellate host, we changed the light intensity of the culture, which led to changes in photosynthetic activity and oxidative stress, and we examined the states of *N. aeruginosum* and the kleptoplast with or without the ingested nuclei.

**Fig. 6:**
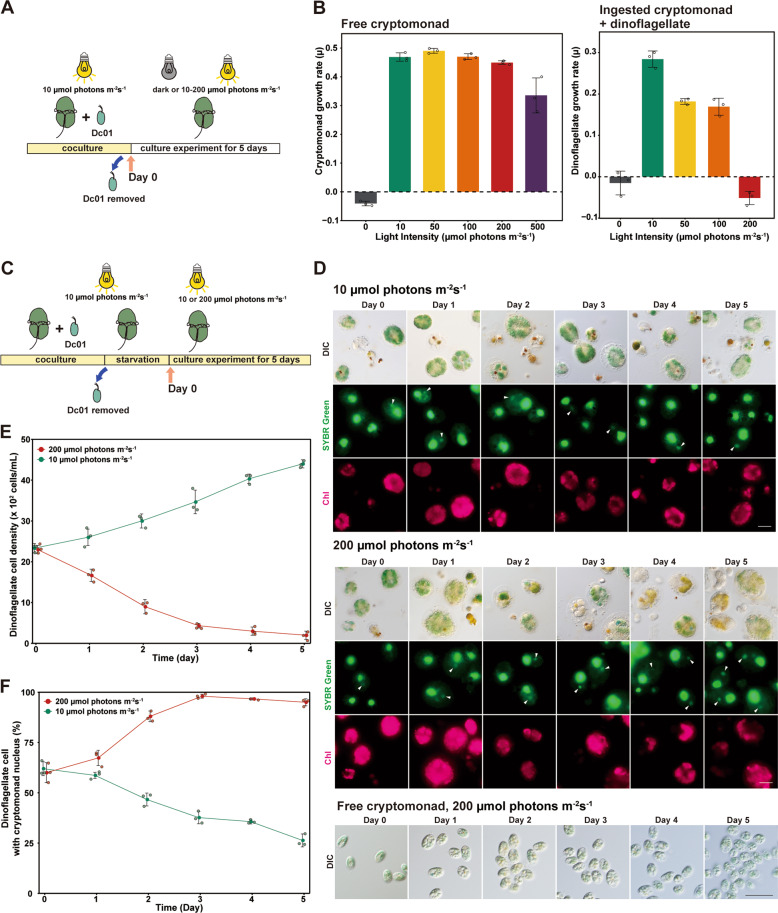
Comparison of growth rate at different light intensities between free *Chroomonas* sp. Dc01 and *N. aeruginosum* with kleptoplasts derived from *Chroomonas* sp. Dc01. **a** To examine the cellular growth of *N. aeruginosum* with kleptoplasts, free *Chroomonas* sp. Dc01 was first removed from the coculture with illumination (10 µmol photons m^−2^ s^−1^) in order to inhibit *N. aeruginosum* from newly ingesting *Chroomonas* sp., and then the culture was exposed to the indicated light intensities. Changes in *N. aeruginosum* cell number for 5 days were determined. Free *Chroomonas* sp. Dc01 alone was simultaneously cultured in the same inorganic medium with illumination (10 µmol photons m^−2^ s^−1^) and then exposed to the indicated light intensities for 5 days. **b** Growth rates under the indicated light intensities. The growth rate (*µ*) was calculated as the difference between the natural logarithm of the cell number (*N*) at different time points (*t*, day 0–5) according to the following equation: *µ* = ln(*N*_t5_/*N*_t0_)/(*t*_5_−*t*_0_). The error bars represent standard deviation (three independent cultures). *P < 0.05; N.S., no significant difference (*t*-test). The changes in respective cell numbers are show in Supplementary Fig. 1. **c** To examine the effects of nuclei derived from *Chroomonas* sp. Dc01 on the growth/survival of *N. aeruginosum* in **e** and **f**, free *Chroomonas* sp. Dc01 was removed from the coculture with illumination (10 µmol photons m^−2^ s^−1^). *N. aeruginosum* with kleptoplasts were further cultured for 12 days until ~40% of the cells had lost nuclei derived from *Chroomonas* sp. Dc01. Then, from hour 0, the culture was kept at 10 µmol photons m^−2^ s^−1^ or subjected to 200 µmol photons m^−2^ s^−1^, and further cultured for 5 days. **d** Micrographs showing the change in the nucleus and kleptoplast derived from *Chroomonas* sp. in *N. aeruginosum* cells at 10 and 200 µmol photons m^−2^ s^−^1, respectively. Cells were stained with SYBR Green. Images of differential interference contrast (DIC), SYBR Green staining, and kleptoplast red fluorescence (Chl) are shown. The arrowhead indicates a nucleus derived from *Chroomonas* sp. For comparison, *Chroomonas* sp. Dc01 cells alone, which were cultured at 200 µmol photons m^−2^ s^−1^, are also shown. Scale bar = 10 μm. Changes in *N. aeruginosum* cell density (**e**) and ratio of *N. aeruginosum* cells possessing the nuclei derived from *Chroomonas* sp. Dc01 (**f**) at 10 and 200 µmol photons m^−2^ s^−1^. The error bars represent standard deviation (three independent cultures).

We first compared the cellular growth rate in the culture of *Chroomonas* sp. alone with that in *N. aeruginosum* cells that had mostly retained *Chroomonas* sp. nuclei (immediately after free *Chroomonas* sp. cells were removed from the coculture with *N. aeruginosum*) under various light intensities: in the dark or in the light at 10, 50, 100, or 200 µmol photons m^−2^ s^−1^ (Fig. 6a, b). In the dark, neither free *Chroomonas* sp. nor *N. aeruginosum* that had ingested *Chroomonas* sp. grew (Fig. 6a, b), which shows that *N. aeruginosum* growth requires light and is based on photosynthesis by *Chroomonas* sp. The growth rate of *Chroomonas* sp. peaked at 50 µmol photons m^−2^ s^−1^ and decreased gradually as light intensity increased further (Fig. 6b; Supplementary Fig. 1). In contrast, the growth rate of *N. aeruginosum* with ingested *Chroomonas* sp. nuclei peaked at 10 µmol photons m^−2^ s^−1^ and substantially decreased at 50 and 100 µmol photons m^−2^ s^−1^; at 200 µmol photons m^−2^ s^−1^, the cell number of *N. aeruginosum* decreased further (Fig. 6b; Supplementary Fig. 1).

We also examined cellular growth/survival at either 10 or 200 µmol photons m^−2^ s^−1^ in an *N. aeruginosum* culture in which ~40% of the cells had lost *Chroomonas* sp. nuclei (Fig. 6c–f). At 10 µmol photons m^−2^ s^−1^, cells without *Chroomonas* sp. nuclei survived for 5 days (Fig. 6c–f). In contrast, at 200 µmol photons m^−2^ s^−1^, the number of cells decreased gradually, while the ratio of cells with *Chroomonas* sp. nuclei increased during 5 days (Fig. 6c–f). Thus, the cells that had lost *Chroomonas* sp. nuclei apparently died more quickly than those that had retained the nuclei.

By microscopic observation, we found that kleptoplasts gradually turned from green to yellow in *N. aeruginosum* cultures under 200 but not 10 µmol photons m^−2^ s^−1^ regardless of the presence or absence of *Chroomonas* sp. nuclei; in contrast, the chloroplast of free *Chroomonas* sp. remained green (Fig. 6d). The rate of cell death, i.e., the decrease in cell number, in *N. aeruginosum* was higher at 200 µmol photons m^−2^ s^−1^ than in the dark (i.e., without photosynthesis), which suggests that cell death under high light is not solely due to the reduced photosynthetic activity of the kleptoplast but also cellular damage in *N. aeruginosum* cells.

These results suggest that (1) high-intensity light conditions, specifically those in the range in which free *Chroomonas* sp. can maintain the integrity of chloroplasts and grow, cause lethal damage to kleptoplasts and *N. aeruginosum* cells and that (2) the ingested *Chroomonas* sp. nucleus increases the longevity of the kleptoplast and its host *N. aeruginosum* under high light conditions.

## Discussion

### Polyploidization and the loss of transcriptional regulation in endosymbionts are common in endosymbiotic evolution

Our previous study showed that *N. aeruginosum* enlarged the kleptoplast only during the retention of the symbiont nucleus [20]. Here, we have shown that the ingested *Chroomonas* sp. nucleus is transcriptionally active in *N. aeruginosum*. Following ingestion, contigs annotated as genes involved in metabolism, including photosynthesis and protein synthesis, were upregulated, while those involved in sensory systems and cellular activities, such as signal transduction and cell motility, were downregulated (Fig. 2; Supplementary Table 1). A similar transcriptomic change has been observed in the cryptomonad nucleus after ingestion by *M. rubrum*, although kleptoplasty was acquired and evolved in the ancestor of this ciliate, independently from that of *Nusuttodinium* spp. [18, 46, 47]. These results suggest that the cryptomonad cell is enslaved as a “machine” specialized for photosynthetic metabolism and protein synthesis that supports growth of the kleptoplast and dinoflagellate host.

Our results also showed that transcriptomic changes that followed illumination were almost abolished in the ingested cryptomonad nucleus. In the kleptoplasty of *N. aeruginosum*, the cryptomonad loses the plasma membrane after being ingested and its cytosol, therefore, becomes enclosed in the phagosome of the dinoflagellate host [19, 20]; presumably, this keeps the cryptomonad cytosol in a more stable condition than if it were exposed to a natural water environment across cell membrane. In addition, the eyespot and flagella of the cryptomonad disappear after it is ingested [19, 20, 48]. Thus, it is apparently reasonable that, after being ingested by the dinoflagellate, the cryptophyte genes involved in sensory systems and cell motility are downregulated and their transcriptomic responses are abolished.

**Fig. 7:**
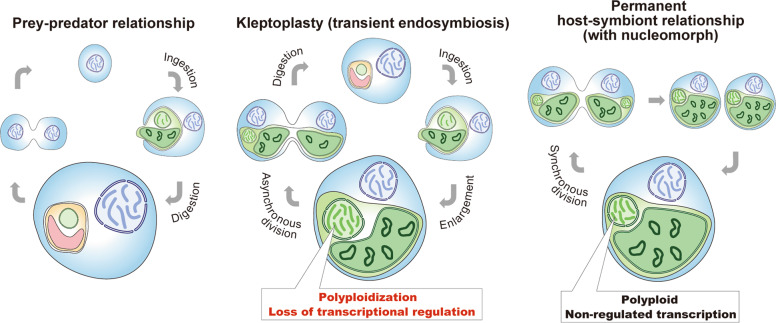
Schematic comparison of prey–predator, transient endosymbiotic (kleptoplasty), and permanent endosymbiotic relationships. Predators start digesting algae immediately after phagocytic ingestion of algae (left). Kleptoplastic species retain the ingested algae for days to weeks in the cell before digesting them in some cases, including that of *N. aeruginosum*, with the nucleus of the algae (middle). The present study shows that the ingested nucleus is polyploidized in the host cell, which increases mRNA levels. The ingested nucleus almost loses transcriptional regulation. These changes were also common to the course of establishment of nucleomorph and chloroplast (also other types of plastids) as obligate endosymbionts in eukaryotes (right).

Although the responses of ingested nuclei to environmental changes have not yet been examined in other kleptoplastic organisms, the loss of the transcriptional response observed in our study is likely to be a common evolutionary trend in endosymbiotic enslavement of endosymbionts by eukaryotic hosts (Fig. 7). In the cryptophyte *Guillardia theta*, cell-cycle-regulated genes, which are specifically transcribed at certain cell-cycle stages in free-living eukaryotes, are constantly expressed from a nucleomorph (a descendant of a red algal endosymbiont) regardless of its replication or division cycle [49]. In the chlorarachniophyte *Bigelowiella natans*, the mRNA levels of almost all of the genes of the nucleomorph (a descendant of a green algal endosymbiont) remained constant throughout a 12-h-light and 12-h-dark cycle [50]. The loss or reduction of the transcriptional response to light is also applicable to endosymbiotic evolution of organelles from cyanobacterial endosymbionts. In contrast to cyanobacteria, chloroplast-encoded transcripts in the green alga *Chlamydomonas reinhardtii* exhibited little change following illumination; therefore, it was suggested that levels and activities of chloroplast-encoded proteins are instead regulated post-transcriptionally [51]. The rhizarian amoeba *Paulinella chromatophora* possesses cyanobacterium-derived photosynthetic organelles known as chromatophores, which were established much more recently than chloroplasts. As is the case in the chloroplast genome, mRNA levels of chromatophore-encoded genes change little following illumination, which suggests that the chromatophore has lost cyanobacterial transcriptional regulation of the response to light [52].

Following ingestion by the dinoflagellate, the ploidy of the cryptomonad nuclear and organellar genomes increased, which correlates with an increase in mRNA levels of the encoded genes (Fig. 4). Our previous study showed that nuclear division ceased in the cryptomonad after it was ingested by the dinoflagellate [19, 20]. In our present study, transcriptome analyses showed that, after ingestion, genes involved in nuclear DNA replication were upregulated, while genes encoding tubulin, which forms spindles for chromosome segregation and an M-phase-protein, CDC20, were downregulated (Fig. 2). These results suggest that the ingested nucleus is arrested in the S phase and continues to be replicated.

In a previous study, although ploidy was not examined, cryptomonad nuclei ingested by the ciliate *M. rubrum* were shown to have been enlarged by up to four times their original diameter after ingestion [53]. The nucleomorph genomes are diploid in the chlorarachniophyte *B. natans* and tetraploid in the cryptophyte *G. theta*, respectively, and it has been suggested that these genomes were polyploidized during endosymbiotic evolution [54]. Chloroplast and mitochondrial genomes also multiplied substantially during endosymbiotic evolution [54, 55]. In addition, the genome of symbiotic or parasitic bacteria such as *Buchnera* [56], *Neisseria* [57], *Borrelia* [58], and *Epulopiscium* [59] are polyploid. Thus, polyploidization of the genomes of endosymbionts/parasites is apparently a common trend in endosymbiotic/parasitic relationships (Fig. 7). Given that the dinoflagellate host is able to divide and inherit kleptoplasts during cell division and that the ingested nucleus is required to grow the kleptoplast [20], if the replicated chromosomes can be segregated and inherited (amitotically because of the loss of tubulin expression) by both daughter cells, the period of kleptoplast retention will be extended. Indeed, in the dinoflagellate *Durinskia baltica*, which contains a permanent diatom endosymbiont, the nucleus of the diatom undergoes amitosis and is inherited by daughter dinoflagellate cells during cell division [15]. Furthermore, the nucleomorph apparently divides amitotically [60–62].

### Comparison of the efficiency of kleptoplasty in the dinoflagellate *N. aeruginosum* and in other organisms

In comparison to the growth of the free-living cryptomonad, the growth rate of *N. aeruginosum* with kleptoplasts was saturated at lower light intensities, even when the nucleus of the cryptomonad had been retained (Fig. 6). Even under low light, genes involved in ROS dissipation were upregulated in the cryptomonad nucleus after its ingestion by the dinoflagellate (Supplemental Table 1). With high-intensity light in the range that did not affect the growth and integrity of the free-living cryptomonad’s chloroplast, we found that the kleptoplast was bleached. These results suggest that the dinoflagellate cell is unable to maximize the potential of the cryptomonad chloroplast. In a similar manner, in another dinoflagellate species that possesses a kleptoplast derived from a haptophyte, the photosynthetic activity of the haptophyte was reduced after it was ingested by the dinoflagellate [63].

In contrast, in *M. rubrum*, the photosynthetic activity of the cryptomonad per chlorophyll is maintained after ingestion; additionally, by enlargement and division of the kleptoplast in the *M. rubrum* cell, the photosynthetic activity per *M. rubrum* cell increases after the ingestion of the cryptomonad [64]. In a photosymbiont-harboring radiolarian species, an increase in the electron transfer rate of photosystem II in a haptophyte was observed after ingestion [65]. These organisms are planktonic and often form blooms around the surface of the sea where they are exposed to high light [66–68]. Thus, these acquired phototrophic organisms have likely acquired the ability to use kleptoplasts/photosymbionts under high light and thereby cope with photosynthetic oxidative stress. Indeed, *M. rubrum* has been shown to produce mycosporine-like amino acids [64], which function as UV radiation protectors and antioxidants [69]. In addition, a haptophyte ingested by a radiolarian has been shown to produce dimethylsulfoniopropionate, which also functions as an antioxidant, at levels more than 100-fold greater than those produced by the free-living algae [68, 70].

In contrast to *M. rubrum* and the radiolarian, *N. aeruginosum* is benthic [48]. Thus, it appears reasonable that *N. aeruginosum* is relatively weak to high light and photosynthetic oxidative stress; however, it is currently unclear whether it produces the antioxidants required to cope with oxidative stress in the light. *P. chromatophora* is also benthic, its chromatophore is bleached, and the cell dies even under moderate light [52]. Therefore, a correlation apparently exists between the habitat of organisms and their ability to use kleptoplasts or photosynthetic organelles; further studies are required to confirm this hypothesis.

## Supplementary information

Supplementary Fig. 1

Supplementary Table 1

Supplementary Table 2

Supplementary Table 3

supplementary figure/table legends

## Data Availability

All of the RNA-seq raw data obtained in this study and the assembled contig sequences were deposited at the DDBJ Sequence Read Archive (accession codes BioProject PRJDB9674) and at the DDBJ Transcriptome Shotgun Assembly Database (accession codes ICPR01000001-ICPR01070969).
