# Supplementary figures and images for: Changes in the transcriptome, ploidy, and optimal light intensity of a cryptomonad upon integration into a kleptoplastic dinoflagellate
Source: ISME J. 2020 Jun 8;14(10):2407–23. doi: 10.1038/s41396-020-0693-4 (PMC7490267; doi:10.1038/s41396-020-0693-4)

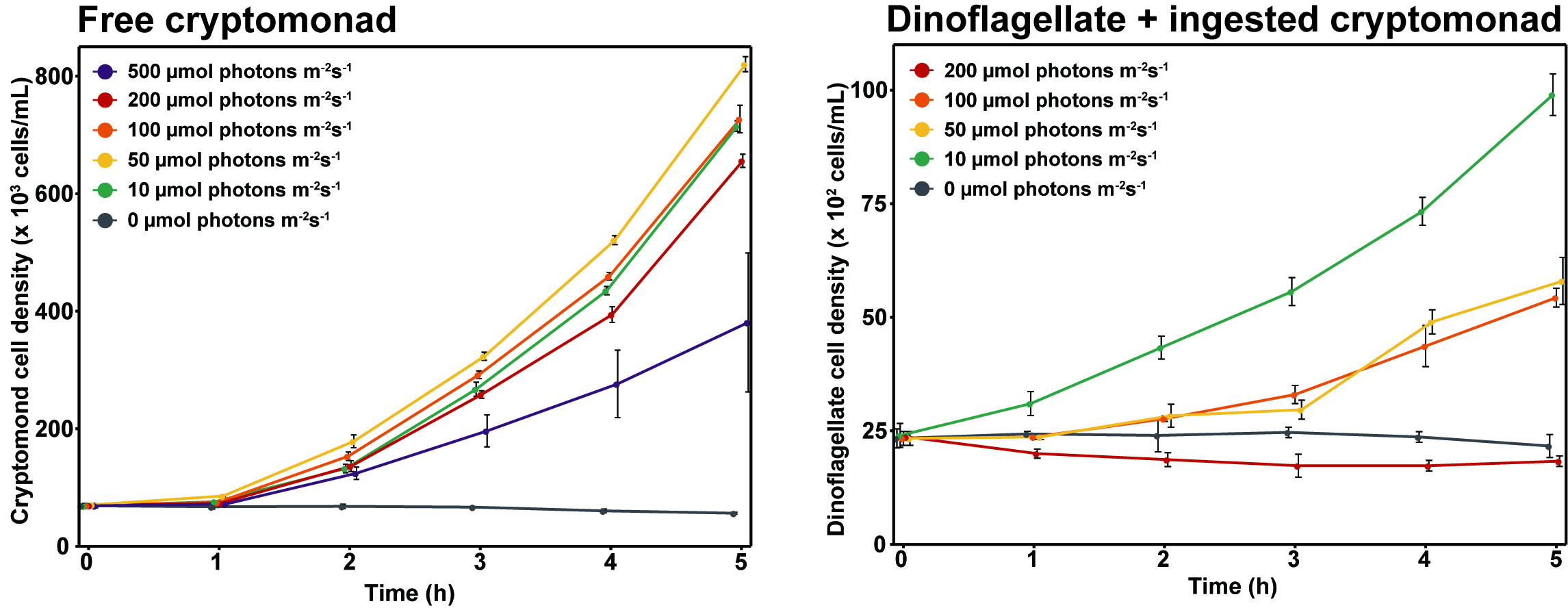

Supplement: Supplementary file 1 — Supplementary Fig. 1 [file 41396_2020_693_MOESM1_ESM.tif]
