# Supplementary material for: Changes in the transcriptome, ploidy, and optimal light intensity of a cryptomonad upon integration into a kleptoplastic dinoflagellate
Source: ISME J. 2020 Jun 8;14(10):2407–23. doi: 10.1038/s41396-020-0693-4 (PMC7490267; doi:10.1038/s41396-020-0693-4)
Supplement: Supplementary file 5 — supplementary figure/table legends [file 41396_2020_693_MOESM5_ESM.docx]

**Supplementary Fig. 1** Changes in cell density in the culture of *N. aeruginosum* with *Chroomonas* sp. Dc01-derived kleptoplasts and that of free *Chroomonas* sp. Dc01 alone. The details are described in Fig. 6A and B. The error bars represent the standard deviation (three independent cultures).

**Supplementary Table 1** KEGG annotations and edgeR output values of *Chroomonas* sp. Dc01 contigs used for DEG (free vs. ingested *Chroomonas* sp.) analysis in Fig. 2. N/A indicates contigs that could not be annotated by the KAAS pipeline.

**Supplementary Table 2** KEGG annotations and log2-fold changes (against values at hour 0) of free-living *Chroomonas* sp. Dc01 DEGs (hour 0 in the dark vs. other time points after illumination). The corresponding values of *Chroomonas* sp. Dc01 ingested by *N. aeruginosum* are also shown. N/A indicates contigs that could not be annotated by the KAAS pipeline.

**Supplementary Table 3** Primers used for qPCR and qRT-PCR.
